# Supplementary material for: Association between Fruit Consumption and Lipid Profile among Children and Adolescents: A National Cross-Sectional Study in China
Source: Nutrients. 2021 Dec 24;14(1):63. doi: 10.3390/nu14010063 (PMC8746805; doi:10.3390/nu14010063)
Supplement: Supplementary file 1 [file nutrients-14-00063-s001.zip › nutrients-1474179-supplementary.pdf]

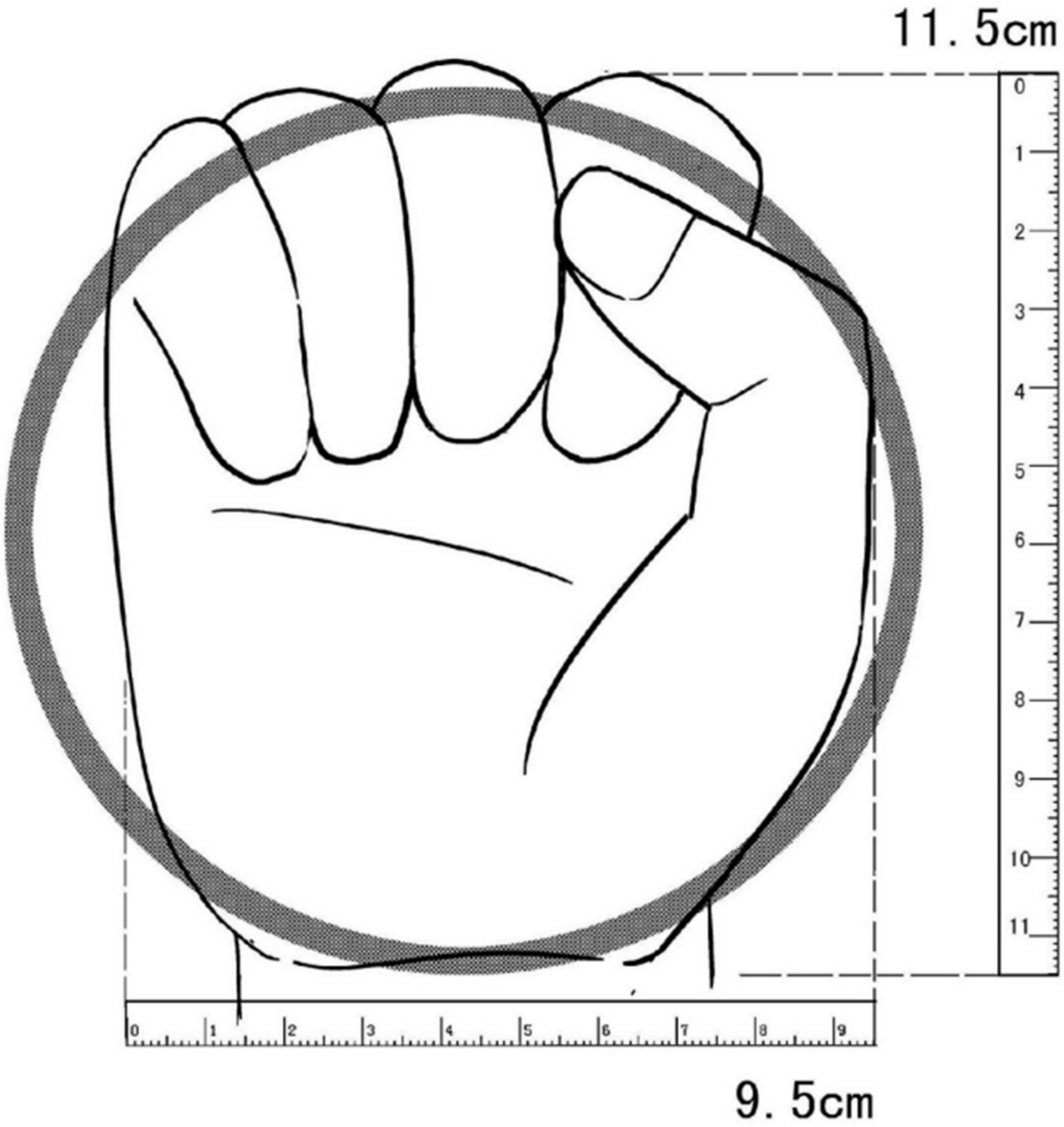

Figure S1. The size of one serving of fruit or vegetable (as the size of a fist of ordinary adult).

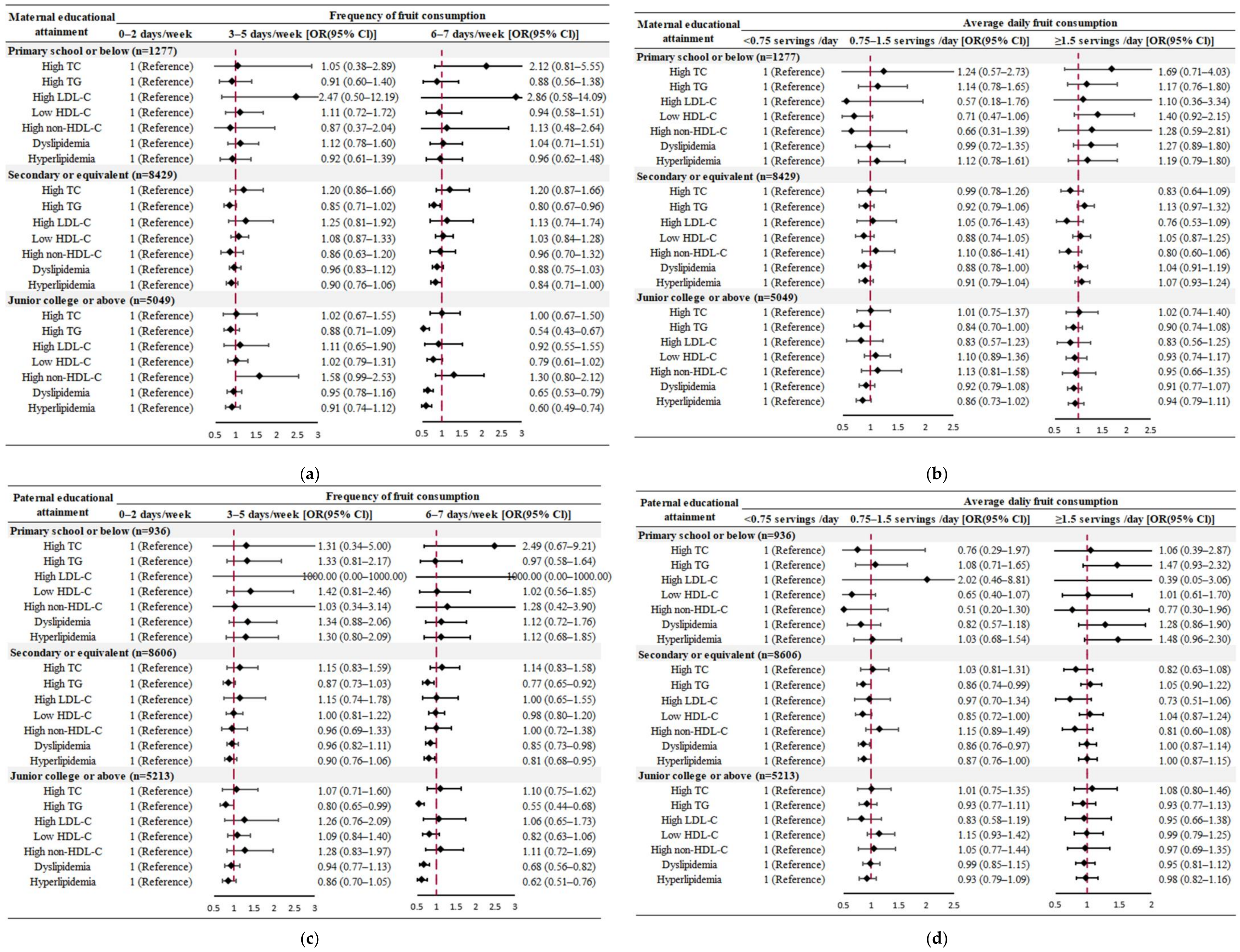

Figure S2. Parental educational attainment-specific analysis of the associations between fruit consumption and lipid profile (adjusted for age, residence area, sex, BMI values, ethnicity, incomes, parental weight, vegetable consumption, sugar-sweetened beverages consumption, meat consumption and physical activity) (**a**) Maternal educational attainment-specific analysis for frequency consumption; (**b**) Maternal educational attainment-specific analysis for average daily consumption; (**c**) Paternal educational attainment-specific analysis for frequency consumption; (**d**) Paternal educational attainment-specific analysis for average daily consumption. TC, total cholesterol; TG, triglycerides; LDL-C, low density lipoprotein-cholesterol; HDL-C, high density lipoprotein-cholesterol; diamond symbol, point estimates; red dashed lines, invalid line.

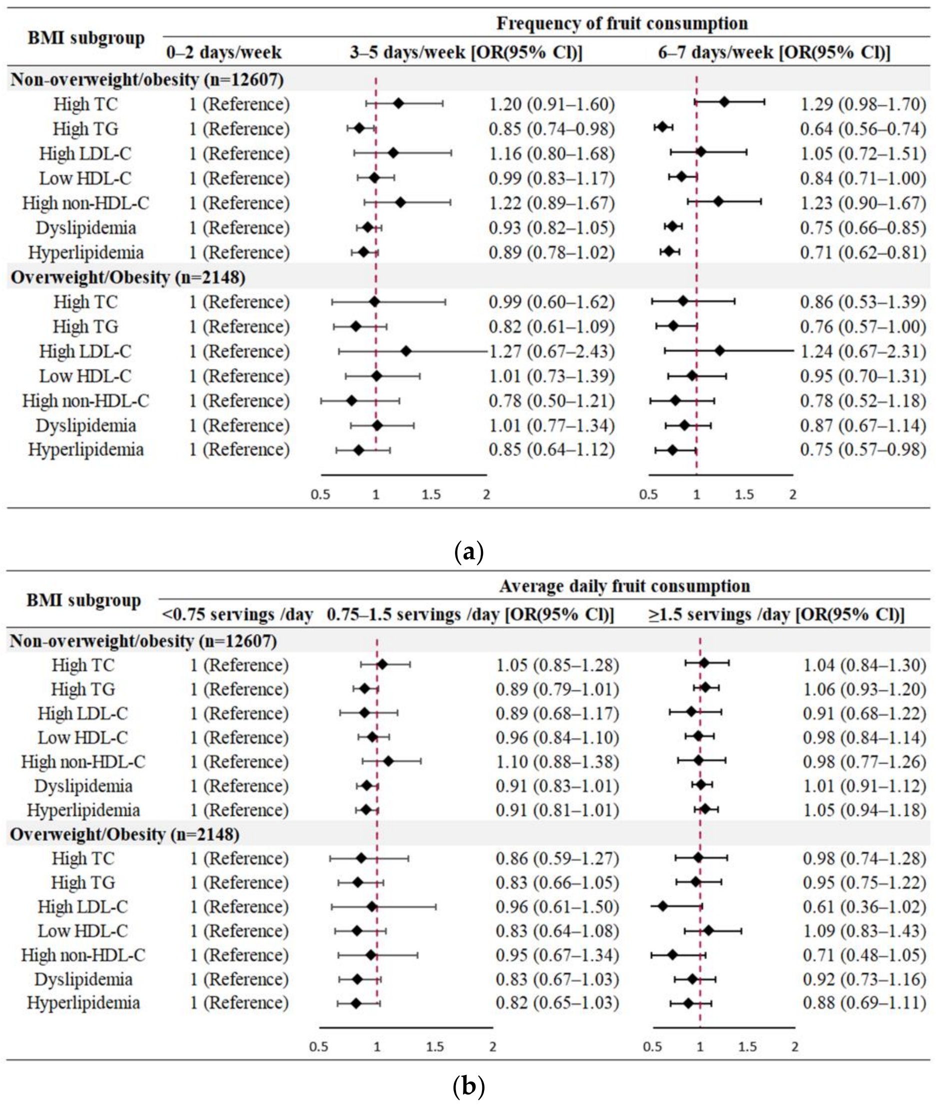

Figure S3. BMI-specific analysis of the associations between fruit consumption and lipid profile (adjusted for age, residence area, sex, ethnicity, incomes, parental weight, parental educational attainment, vegetable consumption, sugar-sweetened beverages consumption, meat consumption and physical activity) (a) BMI-specific analysis for frequency consumption; (b) BMI-specific analysis for average daily consumption. TC, total cholesterol; TG, triglycerides; LDL-C, low density lipoprotein-cholesterol; HDL-C, high density lipoprotein-cholesterol; diamond symbol, point estimates; red dashed lines, invalid line.

Table S1. Prevalence of the abnormal lipid profile (n, %).

Lipid profile	Frequency of fruit consumption				Average daily fruit consumption			
	**0–2 days/week**	**3–5 days/week**	**6–7 days/week**	Significant group comparisons*	**<0.75 servings /day**	**0.75–1.5 servings /day**	**≥1.5 servings /day**	Significant group comparisons*
Total (n=14755)	n=2228	n=5698	n=6829		n=4791	n=5432	n=4532	
High TC	100 (4.49%)	294 (5.16%)	407 (5.96%)	NS	255 (5.32%)	303 (5.58%)	243 (5.36%)	NS
High TG	526 (23.61%)	1226 (21.52%)	1311 (19.20%)	0–2 days/week > 6–7 days/week; 3–5 days/week > 6–7 days/week	990 (20.66%)	1050 (19.33%)	1023 (22.57%)	over 1.5 servings /day > 0.75–1.5 servings /day
High LDL-C	55 (2.47%)	170 (2.98%)	224 (3.28%)	NS	150 (3.13%)	166 (3.06%)	133 (2.93%)	NS
Low HDL-C	330 (14.81%)	779 (13.67%)	835 (12.23%)	0–2 days/week > 6–7 days/week	650 (13.57%)	675 (12.43%)	619 (13.66%)	NS
High non-HDL-C	97 (4.35%)	256 (4.49%)	347 (5.08%)	NS	222 (4.63%)	275 (5.06%)	203 (4.48%)	NS
Dyslipidemia	751 (33.71%)	1877 (32.94%)	2064 (30.22%)	0–2 days/week > 6–7 days/week; 3–5 days/week > 6–7 days/week	1528 (31.89%)	1650 (30.38%)	1514 (33.41%)	over 1.5 servings /day > 0.75–1.5 servings /day
Hyperlipidemia	592 (26.57%)	1437 (25.22%)	1592 (23.31%)	0–2 days/week > 6–7 days/week	1169 (24.40%)	1256 (23.12%)	1196 (26.39%)	over 1.5 servings /day > 0.75–1.5 servings /day
Boys (n=7420)	n=1312	n=2882	n=3226		n=2504	n=2658	n=2258	
High TC	51 (3.89%)	151 (5.24%)	162 (5.02%)	NS	120 (4.79%)	144 (5.42%)	100 (4.43%)	NS
High TG	270 (20.58%)	568 (19.71%)	569 (17.64%)	NS	457 (18.25%)	467 (17.57%)	483 (21.39%)	over 1.5 servings /day > 0.75–1.5 servings /day
High LDL-C	29 (2.21%)	81 (2.81%)	92 (2.85%)	NS	65 (2.60%)	82 (3.09%)	55 (2.44%)	NS
Low HDL-C	212 (16.16%)	418 (14.50%)	421 (13.05%)	NS	364 (14.54%)	345 (12.98%)	342 (15.15%)	NS
High non-HDL-C	53 (4.04%)	132 (4.58%)	148 (4.59%)	NS	106 (4.23%)	137 (5.15%)	90 (3.99%)	NS
Dyslipidemia	416 (31.71%)	920 (31.92%)	928 (28.77%)	NS	754 (30.11%)	773 (29.08%)	737 (32.64%)	NS
Hyperlipidemia	301 (22.94%)	676 (23.46%)	680 (21.08%)	NS	536 (21.41%)	572 (21.52%)	549 (24.31%)	NS
Girls (n=7335)	n=916	n=2816	n=3603		n=2287	n=2774	n=2274	
High TC	49 (5.35%)	143 (5.08%)	245 (6.80%)	6–7 days/week > 3–5 days/week	135 (5.90%)	159 (5.73%)	143 (6.29%)	NS
High TG	256 (27.95%)	658 (23.37%)	742 (20.59%)	0–2 days/week > 3–5 days/week; 0–2 days/week > 6–7 days/week	533 (23.31%)	583 (21.02%)	540 (23.75%)	NS
High LDL-C	26 (2.84%)	89 (3.16%)	132 (3.66%)	NS	85 (3.72%)	84 (3.03%)	78 (3.43%)	NS
Low HDL-C	118 (12.88%)	361 (12.82%)	414 (11.49%)	NS	286 (12.51%)	330 (11.90%)	277 (12.18%)	NS
High non-HDL-C	44 (4.80%)	124 (4.40%)	199 (5.52%)	NS	116 (5.07%)	138 (4.97%)	113 (4.97%)	NS
Dyslipidemia	335 (36.57%)	957 (33.98%)	1136 (31.53%)	0–2 days/week > 6–7 days/week	774 (33.84%)	877 (31.61%)	777 (34.17%)	NS
Hyperlipidemia	291 (31.77%)	761 (27.02%)	912 (25.31%)	0–2 days/week > 3–5 days/week; 0–2 days/week > 6–7 days/week	633 (27.68%)	684 (24.66%)	647 (28.45%)	over 1.5 servings /day > 0.75–1.5 servings /day

*Chi-square tests with Holm Bonferroni correction was used to account for multiple comparisons. TC, total cholesterol; TG, triglycerides; LDL-C, low density lipoprotein-cholesterol; HDL-C, high density lipoprotein-cholesterol

Table S2. Difference of demographics and lifestyle behaviors among age groups.

Characteristics and behaviors	**5–9 years old (n=5646)**	**10–14 years old (n=5776)**	**15–19 years old (n=3333)**	***P*-value**
BMI, kg/m²	16.78±3.02	19.26±3.64	21.18±3.80	<0.0001
Boys (n,%)	2814 (49.84%)	2999 (51.92%)	1607 (48.21%)	0.002
Residence area (n,%)				0.004
Urban	2935 (51.98%)	3175 (54.97%)	1750 (52.51%)	
Rural	2711 (48.02%)	2601 (45.03%)	1583 (47.49%)	
Frequency of consumption (days per week)				
Fruit	5.10±2.04	4.97±2.10	4.58±2.22	<0.0001
Vegetables	6.06±1.77	5.96±1.87	6.07±1.81	0.002
Sugar-sweetened beverages	1.26±1.47	1.65±1.77	2.21±2.10	<0.0001
Meat	4.93±2.24	4.79±2.27	5.51±2.08	<0.0001
Average daily consumption (servings per day)				
Fruit	1.30±1.09	1.45±1.22	1.15±1.04	<0.0001
Vegetables	1.73±1.34	2.05±1.64	1.80±1.46	<0.0001
Sugar-sweetened beverages	0.31±0.61	0.51±0.94	0.62±0.92	<0.0001
Meat	1.09±1.14	1.32±1.43	1.34±1.32	<0.0001
Frequency of physical activity, day	3.04±2.48	3.60±2.53	3.41±2.56	<0.0001
Average daily physical activity, hours and minutes	0.38±0.81	0.39±0.83	0.30±0.83	<0.0001

Abbreviation: BMI, body mass index.
